# Chondrocyte-derived exosomes promote cartilage calcification in temporomandibular joint osteoarthritis

**DOI:** 10.1186/s13075-022-02738-5

**Published:** 2022-02-14

**Authors:** Qian Liu, Ruoxin Wang, Shujie Hou, Feng He, Yuanjun Ma, Tao Ye, Shibin Yu, Hongwei Chen, Helin Wang, Mian Zhang

**Affiliations:** 1 State Key Laboratory of Military Stomatology & National Clinical Research Center for Oral Diseases & Shaanxi International Joint Research Center for Oral Diseases, Department of Oral Anatomy and Physiology and TMD, School of Stomatology, the Fourth Military Medical University, Xi’an, China; 2 Class 1, Grade 2018, School of Stomatology, Zhengzhou University, Zhengzhou, China; 3 School of Basic Medicine, the Fourth Military Medical University, Xi’an, China; 4 Health Center of 73630 Unit of the Chinese People’s Liberation Army, Fuzhou, China; 5 State Key Laboratory of Military Stomatology & National Clinical Research Center for Oral Diseases, Department of Medical Rehabilitation, School of Stomatology, the Fourth Military Medical University, Xi’an, China

**Keywords:** Temporomandibular joint, Osteoarthritis, Exosome, Cartilage calcification, Chondrocyte

## Abstract

**Backgrounds:**

Abnormal cartilage calcification is one of the pathological changes of temporomandibular joint (TMJ) osteoarthritis (OA). Recent studies have reported that exosomes can regulate the formation of abnormal calcified nodules in diseases including atherosclerosis and chronic kidney disease. However, the influences of chondrocyte-derived exosomes on abnormal cartilage calcification in TMJ OA are still unclear.

**Methods:**

TMJ OA was induced by unilateral anterior crossbite (UAC) for 4, 8, or 12 weeks in rats to observe abnormal calcification in TMJ condylar cartilage and exosome formation. Concomitantly, GW4869, the inhibitor of exosome formation, was locally injected to the TMJ of rats under stimulation of UAC, while the exosomes extracted from primary condylar chondrocytes stimulated with fluid flow shear stress (FFSS) were locally injected to rats TMJ.

**Results:**

Abnormal calcification was enhanced in the degenerative cartilage of TMJ OA in UAC rats, and a large number of exosome-like structures with diameters of 50-150 nm were found in the calcified cartilage together with decreased expression of matrix Gla protein (MGP) and increased expression of CD63, tissue-nonspecific alkaline phosphatase (TNAP) and nucleotide pyrophosphatase/phosphodiesterase-1 (NPP1). After FFSS stimulation, the number of exosomes secreted by chondrocytes and the numbers of calcified nodules were increased in cultured cells, and the protein levels of MGP, TNAP, and NPP1 in exosomes were changed. Inhibition of exosome formation, TNAP, and NPP1 or supplementation with exogenous MGP effectively alleviated FFSS-induced chondrocyte calcification. Local injection of GW4869, the exosome inhibitor, alleviated TMJ OA-related cartilage degeneration and calcification in UAC rats. Local injection of exosomes obtained from chondrocytes stimulated by FFSS to the TMJs of normal rats induced cartilage degeneration and calcification similar to that in TMJ OA.

**Conclusions:**

Abnormal biomechanical loading leads to enhanced formation of chondrocyte-derived exosomes, in which promoters of calcification increased and inhibitors decreased, resulting in accelerating abnormal cartilage calcification in TMJ OA. The inhibition of degenerative chondrocyte-derived exosomes is expected to be a new way to prevent and treat TMJ OA.

**Supplementary Information:**

The online version contains supplementary material available at 10.1186/s13075-022-02738-5.

## Introduction

The temporomandibular joint (TMJ) is one of the most common sites of osteoarthritis (OA). TMJ-OA is the most serious subtype of TMJ disorders (TMD), which is a widespread orofacial problem, causing pain and discomfort in patients [1]. In the Chinese mainland, more than 14% of TMD patients present manifestations of TMJ OA on computed tomography examination [2]. Similar to OA in other joints, cartilage calcification is one of the changes in TMJ OA [3, 4]. Calcium crystals, such as basic calcium phosphate (BCP) and calcium pyrophosphate dihydrate (CPPD) crystals, form calcified nodules, which further stimulate inflammation and matrix degradation in cartilage, aggravating the degeneration of cartilage in OA [5–7]. However, the mechanism of abnormal calcification formation in OA cartilage remains to be determined.

Exosomes are vesicle-like bodies with diameters of 30–100 nm, secreted by living cells [8, 9]. Exosomes contain a wide variety of bioactive substances, such as proteins, mRNA, and miRNA, involved in numerous physiological and pathological processes [10, 11]. Recent studies have demonstrated that exosomes promoted abnormal calcification in diseases including atherosclerosis and chronic kidney disease [12–14]. Chondrocytes, the only cell type in articular cartilage, are crucial in the maintenance of cartilage homeostasis [15, 16]. However, there are few studies on the role of chondrocyte-derived exosomes in OA. Moreover, there was obvious abnormal calcification in degenerative cartilage during TMJ OA, with a large number of exosome-like bilayer vesicles structures in the abnormal calcified matrix around chondrocytes [5, 17], suggesting that exosomes derived from degenerative chondrocyte may be involved in abnormal calcification in TMJ OA cartilage. The hypothesis of the study is that exosomes secreted by chondrocytes under aberrant biomechanical stimulation in TMJ cartilage participate in OA-related abnormal calcification in articular cartilage.

Recently, unilateral anterior crossbite (UAC) stimulation to rats induced degenerative lesions in TMJ condylar cartilage, presented with decreased cartilage thickness, accelerated matrix degradation, and significantly increased OA scores [18–20]. In this study, rats with TMJ OA and primary condylar chondrocytes stimulated with fluid flow shear stress (FFSS) were used to explore whether chondrocyte-derived exosomes under the stimulation of abnormal biomechanics are involved in the regulation of abnormal calcification in OA cartilage.

## Materials and methods

### UAC application

Six-week-old female Sprague–Dawley rats obtained from the Animal Center of the Fourth Military Medical University (FMMU) were subjected to UAC for 4, 8, or 12 weeks randomly. All animal procedures were approved by the Institutional Animal Care Guidelines and approved by the Laboratory Animal Care & Welfare Committee, School of Stomatology of FMMU to minimize animal stresses. All animals were housed in a pathogen-free room and given sterilized food and distilled water during the study. The animals were housed at a density of no more than four animals in a single cage (measuring 50 × 40 × 25 cm) under an ambient temperature of 20 ± 2°C with a humidity of 55% ± 5% and good ventilation and also 12-h/12-h dark/light cycles (4 W per square meter). The sterilized wood chip bedding was replaced every other day. Animal health status was monitored twice daily. All animals were healthy throughout the study. No adverse events other than TMJ pathology were observed. All animals were euthanized via overdose with a single intraperitoneal injection of pentobarbital sodium before tissue harvesting. This study complied with the Animal Research Reporting in Vivo Experiments (ARRIVE) guidelines.

UAC stimulation was applied with a pair of metal prosthetics as we previously reported [5, 18]. Briefly, a small metal tube (length = 2.5 mm, inside diameter = 3 mm) was adhesive onto the left maxillary incisor, and a larger tube (length = 4.5 mm, inside diameter = 3.5 mm) onto the left mandibular incisor. The end of the large tube was bent to create a 135° angle leaning toward the labial side to create a cross-bite relationship between the top and bottom incisors.

### Experimental sample size

For in vivo experiments, rats were sacrificed via overdose intraperitoneal injection of pentobarbital sodium at 4 weeks, 8 weeks, and 12 weeks after UAC stimulation. No significant difference was observed in the manifestation of the OA phenotype between the right and left TMJs in the UAC group [5, 18]. The eighteen-right rat TMJ blocks of each group were randomly used for histochemical and immunohistochemical (IHC) staining (*n* = 6), von Kossa staining (*n* = 6), and transmission electron microscopy (TEM) (*n* = 6), respectively. Every three of eighteen-left TMJ blocks from each group were randomly pooled for RNA extraction and quantitative real-time PCR (qRT-PCR) analyses (*n* = 6). For in vitro experiments, the cell cultures and subsequent procedures were repeated six times for each group (*n* = 6).

### Histochemistry and IHC

TMJs blocks were fixed in 4% paraformaldehyde, decalcified with 4% EDTA for 4 weeks, dehydrated in ethanol, embedded in paraffin, and then cut sagittally into 5-μm-thick sections. The central sagittal sections of each block were selected for safranine O staining (S2255, Sigma-Aldrich, USA) and IHC staining. IHC staining with primary antibodies against CD63 (5 μg/ml, ABIN6259068, Antibodies, Germany), matrix Gla protein (MGP) (5 μg/ml, ab192396, Abcam, UK), tissue-nonspecific alkaline phosphatase (TNAP) (5 μg/ml, ab203106, Abcam, UK), and nucleotide pyrophosphatase/phosphodiesterase-1 (NPP1) (5 μg/ml, ABIN1858719, Antibodies, Germany) was performed as a standard, three-step, avidin-biotin complex staining procedure. Images were captured by a Leica light microscope (Leica 2500, Hessen, Germany).

The assessment of histological parameters was performed as we previously reported [21, 22]. The image of condylar cartilage was divided into three sections with equal width (anterior, middle, and posterior; Supplemental Fig. 1). The analysis of Safranine O (SO) staining, indicating the proteoglycan amount in TMJ cartilage, was performed using Image-Pro Plus 6.0 software. The image of condylar cartilage was divided into three sections with equal width (anterior, middle, and posterior). The integral optical density (IOD) of SO in the whole cartilage layer at the middle and posterior thirds of each section was measured and the average value of the was calculated to represent the IOD of SO staining for each section. In the sections with Von Kossa staining, indicating the calcified cartilage, a region of interest (ROI) at the center of each section was boxed (width: 200 mm, height: cartilage thickness) and analyzed for the thickness of the cartilage and the proportion of calcified cartilage. The values from three ROIs were averaged and reported for each sample. The grade of histomorphology was analyzed with a modified assessment system on OA grade based on the Osteoarthritis Research Society International (OARSI) score system, 0-VI representing the depth of OA progression as we previously reported [19].

### Von Kossa staining

TMJs blocks were fixed in 2.5% glutaraldehyde for 48 h, dehydrated in ethanol, and embedded in a mixture of methyl methacrylate and dibutyl phthalate. Sections (15-mm thickness) were made with a Leica SP1600 hard tissue slicer and stained with von Kossa reagents (GMS80045, Baoman Biotech, China). Images were captured by a Leica light microscope (Leica 2500, Hessen, Germany).

### TEM

Dissected TMJ blocks were fixed in 2.5% glutaraldehyde in phosphate buffer (pH 7.2) for 24 h, decalcified in 10% EDTA for one month, post-fixed in 1% osmium tetroxide for 1 h, dehydrated in ethanol, perfused with propylene oxide, and embedded in Epon 812. Ultrathin sections (40-nm thickness) were made, stained with uranyl acetate and lead citrate. The stained sections were examined under a transmission electron microscope (H-600, Hitachi).

### Cell culture and FFSS application

Primary condylar chondrocytes were isolated from the TMJs of three-week-old female Sprague–Dawley rats (Animal Center, FMMU) as we described previously [23]. Briefly, cartilage was carefully dissected from the condyles of rat TMJs and digested with 0.25% trypsin (T4674, Sigma-Aldrich, USA) for 20 min followed by 0.2% type II collagenase (Gibco, USA) for 3 h. The isolated chondrocytes were cultured at a density of 5×10^3^ cells/cm^3^ in DMEM (Hyclone) with 10% (v/v) FBS and 1% penicillin-streptomycin (Hyclone, USA) on type I collagen-coated glass slides (Flexcell) for 21 days. During 21 days, the chondrocytes were exposed to FFSS (4, 8, and 16 dyn/cm^2^) for 1 hour every three days and the replaced medium was used to extract exosomes. The cultured chondrocytes were used for immunoblot analyses and Alizarin red staining after 21 days. After fixation with 4% paraformaldehyde for 30 min, cultured chondrocytes were washed three times with PBS before staining with Alizarin red (A5533, Sigma-Aldrich, USA) for 5 min. The samples were washed again with PBS before observation.

To study the influence of exosomes on calcification, after 16 dyn/cm^2^ FFSS stimulation, chondrocytes were switched to medium with 5 or 10 μM GW4869 (S7609, Selleck, USA), the exosome inhibitor, 1 μg/ml MGP (ab113156, Abcam, UK), 10 μM SBI-425 (TNAP inhibitor, 2963, Axon, USA), or 10 μM Ennp-1-IN-1 (NPP1 inhibitor, S0501, Selleck, USA). In addition, isolated chondrocytes were also cultured using the medium with 5 or 10 μg/ml exosomes secreted by FFSS-stimulated chondrocytes.

### Exosome isolation

Exosomes were isolated from culture medium via ultracentrifugation [24]. Briefly, the culture medium centrifuged at 300×*g* for 10 min, and the retained supernatant was then centrifuged at 2000×*g* for 10 min. Subsequently, the retained supernatant was subjected to centrifugation at 10,000×*g* for 30 min, 100,000×*g* for 70 min, and collected the pellet. Finally, the pellet was resuspended in PBS and subjected to centrifugation at 100,000×*g* for 70 min, and the pellet was collected for further use. We can obtain about 120–150 μg exosome from about 10^7^ cells.

### TMJ local injections in rats

Fifty microliters GW4869 (10 μM in PBS) was injected into the TMJs of rats under anesthetization from the beginning at the first day of UAC stimulation. Moreover, 50μl exosomes (10 μg/ml in PBS) secreted by condylar chondrocytes under 16 dyn/cm^2^ FFSS stimulation were injected into the TMJs of rats without UAC stimulation under anesthetization. The injections were performed by inserting a 100-μl Hamilton needle below the zygomatic arch between the eye and ear until it reached the posterior mandibular ramus, which was then used as a reference point for repositioning the needle head to the outside of the TMJ for final penetration and then drug delivery as we previously reported [25, 26]. Both TMJs in each animal were injected every other day for 8 weeks or 12 weeks.

### RNA extraction and qPCR

The condylar cartilage of TMJ was carefully dissected from the mandible and kept in -80°C using RNAlater™ Stabilization Solution (AM7020, Invitrogen, USA) until RNA extraction. Upon extraction of RNA, the cartilage blocks were pre-cooled in liquid nitrogen for about 10min in the specially-made container. After pre-cooled, the container was taken out quickly and smashed 2–3 times. Finally, the shattered cartilage blocks were transferred into Tripure (Roche, Switzerland) for subsequent RNA extraction. After the culturation of cells and the isolation of exosome, 500 μl Tripure (Roche, Switzerland) was added in the plates or tubes for extraction of RNA and protein according to the manufacturer’s instructions. The ratio of 260/280 of the extracted RNA is about 1.9-2.0 while the concentration is about 300-800 ng/μl. RNA from each sample was reverse transcribed to cDNA utilizing the PrimeScript^TM^ RT Reagent Kit Perfect Real-Time (RR036A, TaKaRa Biotechnology, Japan). The primers used are shown in Table 1 and Supplemental Table 1. The amount of target cDNA relative to GAPDH cDNA was calculated using the formula 2^−ΔCt^. Each cartilage or cell sample was analyzed in triplicate, and each mean value was normalized to the control group as specified.

**Table 1:** Gene primer sequences of rats used for qRT-PCR

Genes	Forward primer	Reverse primer
Col2a1	GGAGCAGCAAGAGCAAGGAGAAG	TCAGTGGACAGTAGACGGAGGAAAG
Aggrecan	AGAAGAGACCCAAACAGCAGAAACAG	GCAGGTGGCTCCATTCAGACAAG
MMP13	CAAGATGTGGAGTGCCTGATGTGG	GCGTGTGCCAGAAGACCAGAAG
Runx2	CTTCGTCAGCGTCCTATCAGTTCC	TCCATCAGCGTCAACACCATCATTC
Osteocalcin	GGACCCTCTCTCTGCTCACTCTG	ACCTTACTGCCCTCCTGCTTGG
CD63	CGGTGGAAGGAGGAATGAAGTGTG	AACCTGAACTGCTACGCCAATGG
Mgp	CAGCAGAGGTGGCGAGCTAA	ATGGCGTAGCGCTCACACAG
Npp1	AGGGCATTCACACGGACCA	CTTCAGGCCATCCATCAGCA
Tnap	CACGTTGACTGTGGTTACTGCTGA	CCTTGTAACCAGGCCCGTTG
Gapdh	GGCACAGTCAAGGCTGAGAATG	ATGGTGGTGAAGACGCCAGTA

### Immunoblot analyses

The extracted protein samples (20 μg) of each group were separated by SDS-PAGE gels and then transferred to activated PVDF membranes. After blocking in 3% BSA for 1 h, the membranes were incubated with primary antibodies against CD63 (2 μg/ml), MGP (2 μg/ml), TNAP (2 μg/ml), NPP1 (2 μg/ml), and β-actin (1 μg/ml, ab8226, Abcam, UK). The membranes were then incubated with HRP-conjugated IgG secondary antibodies (1 μg/ml, A0216 or A0208, Beyotime Biotechnology, China) and quantified with Image Lab software (version 5.1, Bio-Rad, USA). The protein levels in each sample were analyzed and are reported as the ratios to β-actin or CD63 levels.

### Statistics

The data are presented as the mean ± SD. Statistical analysis was performed using SPSS software, version 11.0. Before comparison, the normality of the data distribution was tested by the Shapiro-Wilk test, and Levene’s test was used to assess the homogeneity of variance. Comparisons between two groups were performed by Student’s t-test. For comparisons among four groups, one-way ANOVA was performed, and it was followed by Tukey’s post hoc tests to evaluate the statistical significance in pairwise comparisons. Statistical significance was defined as p < 0.05.

## Results

### Abnormal calcification in degenerative cartilage from rat with TMJ OA

**Fig. 1:**
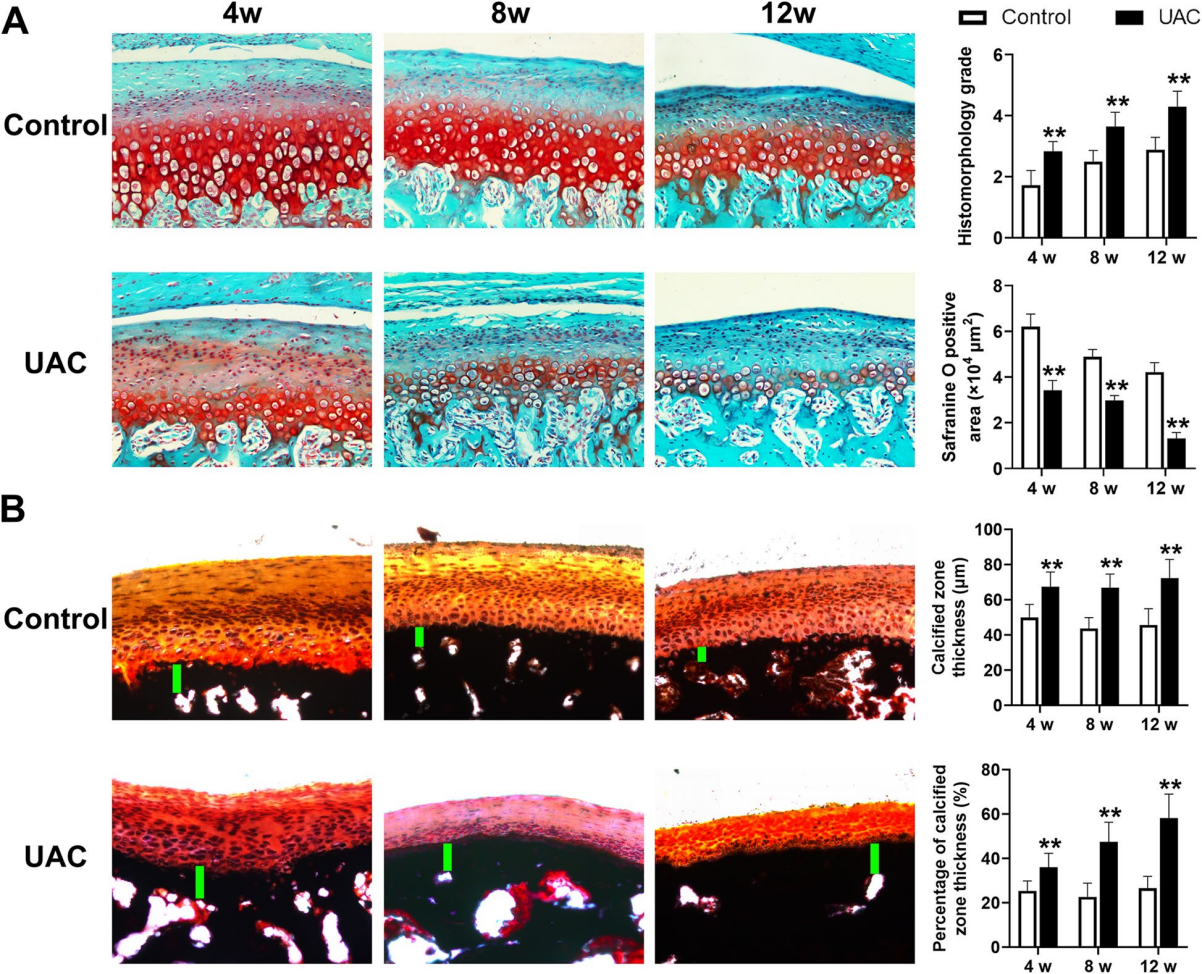
Degeneration and calcification in condylar cartilage of rats with TMJ OA induced by UAC stimulation. **A** Safranine O staining and the grade of histomorphology presented obvious cartilage degeneration was in the TMJs of UAC rats (*n* = 6). **B** Von Kossa staining revealed obvious cartilage calcification was in the TMJs of UAC rats (*n* = 6). Scale bar = 100 μm. Green bar, calcified cartilage. Control, control group; UAC, unilateral anterior crossbite group; 4w, 4 weeks; 8w, 8 weeks; 12w, 12 weeks. All data are presented as means ± SD. The data of compared groups were from the populations of Gaussian distribution and consistent with homogeneity of variance. Comparisons between the control and UAC groups of the same timepoint were performed by Student’s *t* test. **P < 0.01, compared with the age-matched control group

**Fig. 2:**
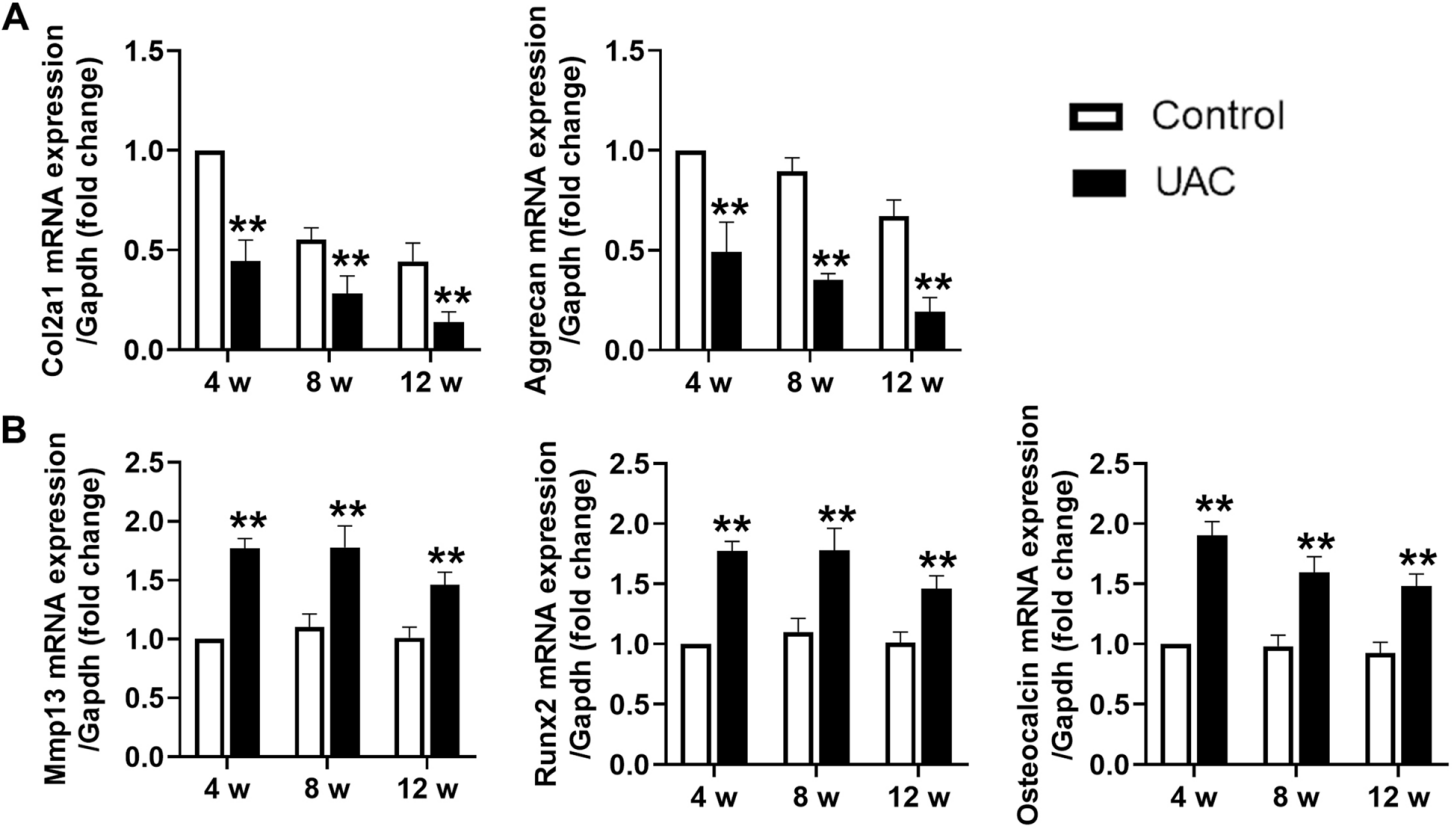
Changed mRNA expression in condylar cartilage of rats with TMJ OA induced by UAC stimulation. **A** mRNA expression of Col2a1 and Aggrecan was significantly decreased in the TMJs of UAC rats (*n* = 6). **B** mRNA expression of Mmp13, Runx2, and Osteocalcin was significantly increased in the TMJs of UAC rats (*n* = 6). Control, control group; UAC, unilateral anterior crossbite group; 4w, 4 weeks; 8w, 8 weeks; 12w, 12 weeks. All data are presented as means ± SD. The data of compared groups were from the populations of Gaussian distribution and consistent with homogeneity of variance. Comparisons between the control and UAC groups of the same timepoint were performed by Student’s *t* test. **P < 0.01, compared with the age-matched control group

In the TMJ condylar cartilage from rats of the control groups, chondrocytes were arranged orderly and cellular layers were clear. Safranine O positive staining was positive in the prehypertrophic layer and hypertrophic layer, indicating abundant proteoglycan in the cartilage matrix. However, in the TMJ condylar cartilage from rats stimulated with UAC, there were cell-free areas and chondrocyte arrangement was disordered. Meanwhile, decreased safranine O-positive area (4 weeks: *P *< 0.01, 8 weeks: *P *< 0.01, 12 weeks: *P *< 0.01) indicated enhanced degradation of cartilage matrix. In addition, the value of the modified OARSI histomorphology score was significantly higher in rats of the UAC group than ones in the control group (4 weeks: *P *< 0.01; 8 weeks: *P *< 0.01, 12 weeks: *P *< 0.01) (Fig. 1A). Von Kossa staining showed that in the control groups, calcification of the condylar cartilage was presented only in the deep hypertrophic layer. The thickness of the calcified layer (4 weeks: *P *< 0.01, 8 weeks: *P *< 0.01, 12 weeks: *P *< 0.01) and the proportion of the calcified layer to the whole cartilage thickness in the condylar cartilage (4 weeks: *P *< 0.01, 8 weeks: *P* < 0.01, 12 weeks: *P *< 0.01) were significantly increased in the UAC groups than that in the control groups (Fig. 1B). Together with the histomorphology changes, the mRNA expression of type-II collagen (Col2a1; 4 weeks: *P *< 0.01, 8 weeks: *P *< 0.01, 12 weeks: *P *< 0.01) and Aggrecan (4 weeks: *P *< 0.01, 8 weeks: *P *< 0.01, 12 weeks: *P *< 0.01) were significantly decreased in the condylar cartilage from rats in the UAC groups (Fig. 2A), while mRNA expression of matrix metalloproteinase-13 (Mmp13; 4 weeks: *P *< 0.01, 8 weeks: *P *< 0.01, 12 weeks: *P *< 0.01), runt-related transcription factor 2 (Runx2; 4 weeks: *P *< 0.01, 8 weeks: *P *< 0.01, 12 weeks: *P *< 0.01) and Osteocalcin (4 weeks: *P *< 0.01, 8 weeks: *P *< 0.01, 12 weeks: *P *< 0.01), markers of chondrocyte de-differentiation, were significantly increased in the condylar cartilage from rats in the UAC groups (Fig. 2B).

### Exosomes increased in degenerative cartilage from TMJ OA rats

**Fig. 3:**
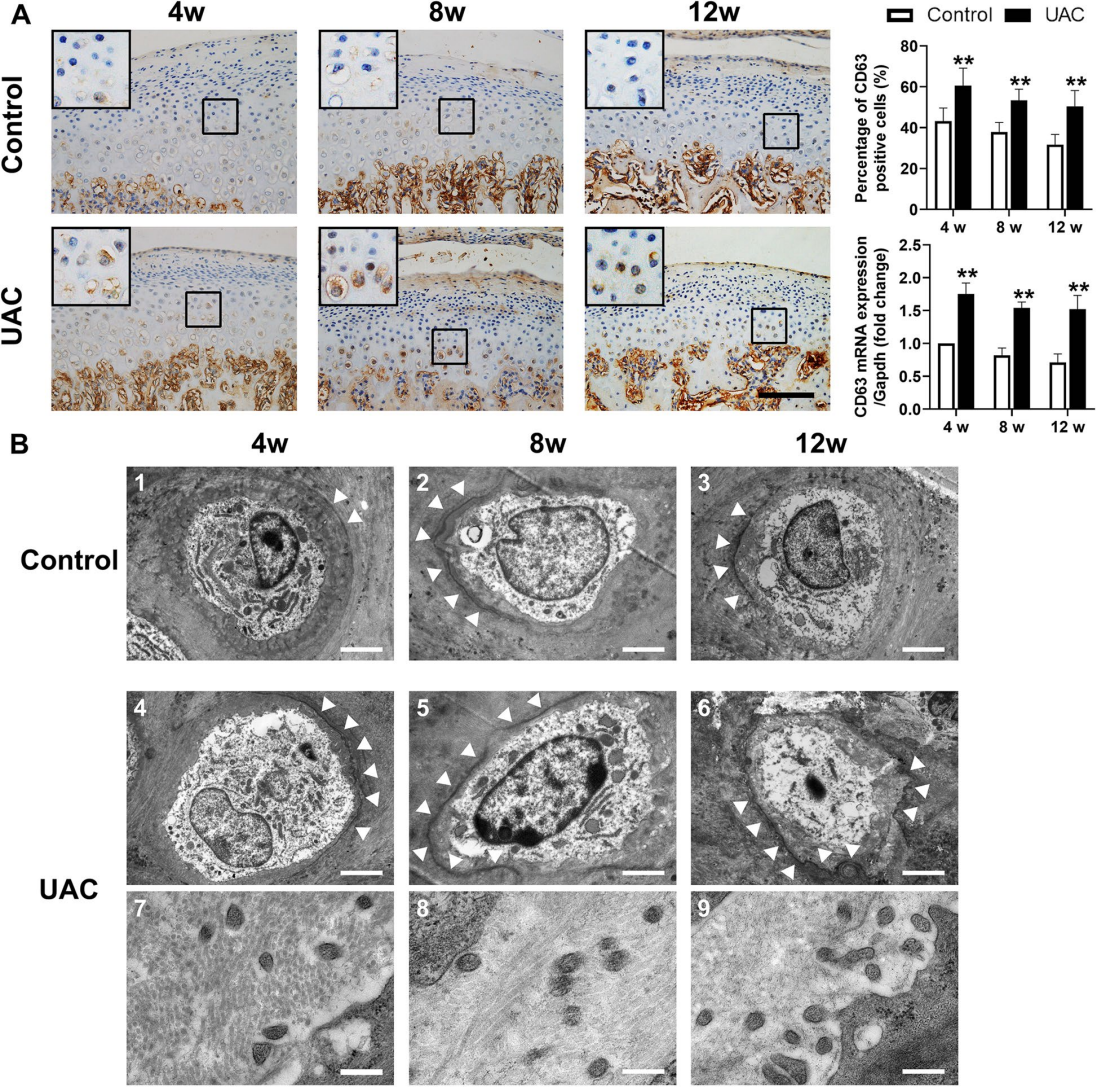
Increased formation of exosomes in the TMJ OA cartilage of UAC rats. **A** The number of CD63-positive chondrocytes and the mRNA expression of CD63 were increased in the condylar cartilage of UAC rats (*n* = 6). Scale bar = 100 μm. All data are presented as means ± SD. The data of compared groups were from the populations of Gaussian distribution and consistent with homogeneity of variance. Comparisons between the control and UAC groups of the same timepoint were performed by Student’s *t* test. **P < 0.01, compared with the age-matched control group. **B** TEM observation of the condylar cartilage in age-matched control and UAC rats. The white arrows in 1–6 pointed out the calcification margin around chondrocytes. One to 3 showed the calcification in the control group and 4–6 showed the calcification in the UAC group while 7–9 showed the increased exosome-like structures in the calcified matrix. Scale bar, 1–6 = 5 μm; 7–9 = 100 nm. Control, control group; UAC, unilateral anterior crossbite group; 4w, 4 weeks; 8w, 8 weeks; 12w, 12 weeks

IHC staining showed that CD63, a marker for exosomes, was mainly in the hypertrophic layer of TMJ condylar cartilage in the control groups. However, in the UAC groups, CD63-positive chondrocytes were also found in the pre-hypertrophic layer of TMJ condylar cartilage. At three time points, the ratios of CD63-positive chondrocytes in the UAC groups were all higher than those in the control groups (4 weeks: *P *< 0.01, 8 weeks: *P *< 0.01, 12 weeks: *P *< 0.01), and the mRNA expression of CD63 was also significantly higher in UAC groups than in the control groups (4 weeks: *P *< 0.01, 8 weeks: *P* < 0.01, 12 weeks: *P *< 0.01) (Fig. 3A). There was less calcification around the hypertrophic-layer chondrocytes of the TMJ condylar cartilage in the control groups than in the UAC groups indicated by TEM images (1–3 of control groups compared with 4–6 of UAC groups in Fig. 3B). In addition, a large number of exosome-like structures with diameters of 50–150 nm were observed in the surrounding cartilage matrix (7–9 in Fig. 3B).

**Fig. 4:**
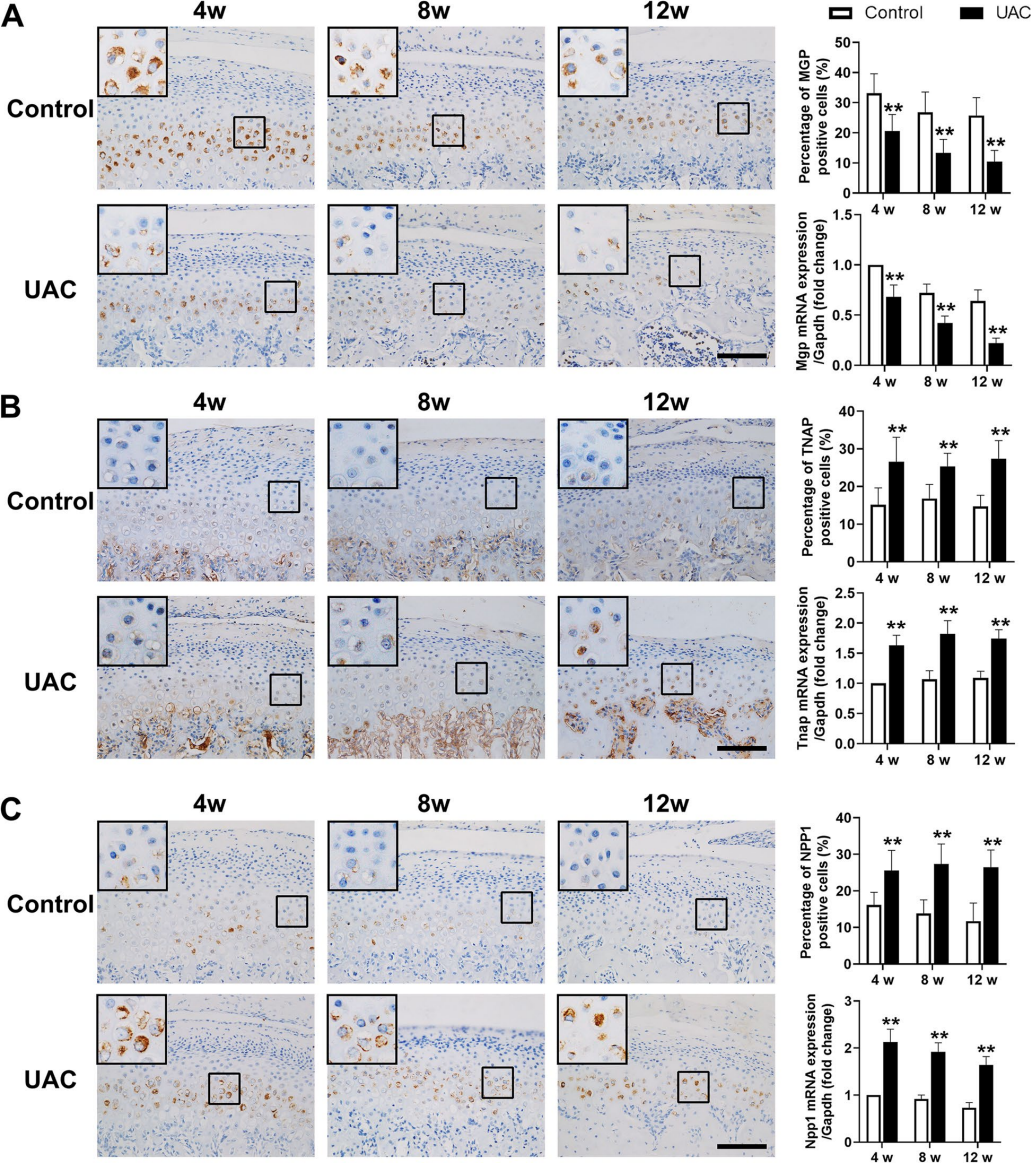
The expression of calcification inhibitor was decreased in condylar cartilage, while the expression of calcification promotors was increased. **A** The number of MGP-positive chondrocytes and the mRNA expression of Mgp were decreased in the condylar cartilage of UAC rats (*n* = 6). **B** The number of TNAP-positive chondrocytes and the mRNA expression of Tnap were decreased in the condylar cartilage of UAC rats (*n* = 6). **C** The number of NPP1-positive chondrocytes and the mRNA expression of Npp1 were decreased in the condylar cartilage of UAC rats (*n* = 6). Scale bar = 100 μm. Control, control group; UAC, unilateral anterior crossbite group; 4w, 4 weeks; 8w, 8 weeks; 12w, 12 weeks. All data are presented as means ± SD. The data of compared groups were from the populations of Gaussian distribution and consistent with homogeneity of variance. Comparisons between the control and UAC groups of the same timepoint were performed by Student’s *t* test. **P < 0.01, compared with the age-matched control group

MGP is an inhibitor of calcification, while TNAP and NPP1 are enzymes that promote calcification. The ratios of MGP-positive chondrocytes (4 weeks: *P *< 0.01, 8 weeks: *P *< 0.01, 12 weeks: *P *< 0.01) and the mRNA expression of Mgp (4 weeks: *P *< 0.01, 8 weeks: *P *< 0.01, 12 weeks: *P *< 0.01) significantly decreased in the condylar cartilage from rats in the UAC groups (Fig. 4A). The ratios of TNAP-positive and NPP1-positive chondrocytes (TNAP, 4 weeks: *P *< 0.01, 8 weeks: *P *< 0.01, 12 weeks: *P *< 0.01; NPP1, 4 weeks: *P *< 0.01, 8 weeks: *P *< 0.01, 12 weeks: *P *< 0.01) and the mRNA expression of Tnap and Npp1 (Tnap, 4 weeks: *P *< 0.01, 8 weeks: *P *< 0.01, 12 weeks: *P *< 0.01; Npp1, 4 weeks: *P *< 0.01, 8 weeks: *P *< 0.01, 12 weeks: *P *< 0.01) were significantly increased in the condylar cartilage from rats in the UAC groups (Fig. 4B and C).

### FFSS promoted exosome formation and calcification in primary condylar chondrocytes

**Fig. 5:**
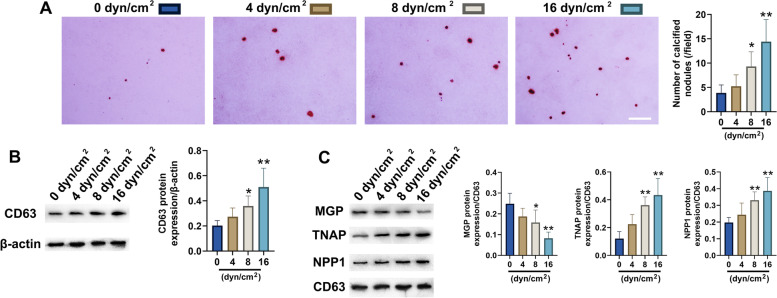
FFSS promoted calcification and exosome formation in cultured condylar chondrocytes and changed the protein levels in exosomes. **A** FFSS promoted the formation of calcified nodules in cultured condylar chondrocytes (*n* = 6). Scale bar = 100 μm. **B** FFSS promoted the exosome formation of cultured condylar chondrocytes (*n* = 6). **C** FFSS changed the protein levels in exosomes, as demonstrated by decreased expression of MGP and increased expression of TNAP and NPP1 (*n* = 6). All data are presented as means ± SD. The data of compared groups were from the populations of Gaussian distribution and consistent with homogeneity of variance. Comparisons among groups were performed by one-way ANOVA, and it was followed by Tukey’s post hoc tests to evaluate the statistical significance in groups with the control group (0 dyn/cm^2^ group) respectively. *P < 0.05, **P < 0.01, compared with the control group (0 dyn/cm^2^ group)

To mimic the abnormal biomechanical environment of TMJ OA in vitro, primary condylar chondrocytes were stimulated with FFSS. Alizarin red staining showed that with elevated value of FFSS, the numbers of calcified nodules in primary condylar chondrocytes significantly increased (8 dyn/cm^2^: *P *< 0.05, 16 dyn/cm^2^: *P *< 0.01) (Fig. 5A), accompanied with raised CD63 expression (8 dyn/cm^2^: *P *< 0.05, 16 dyn/cm^2^: *P *< 0.01) (Fig. 5B). In addition, MGP in exosomes secreted by condylar chondrocytes decreased significantly with elevated FFSS value (8 dyn/cm^2^: *P *< 0.05, 16 dyn/cm^2^: *P *< 0.01), while TNAP and NPP1 increased significantly (TNAP, 8 dyn/cm^2^: *P *< 0.01, 16 dyn/cm^2^: *P *< 0.01; NPP1, 8 dyn/cm^2^: *P *< 0.01, 16 dyn/cm^2^: *P *< 0.01) (Fig. 5C).

### Inhibition of exosome formation alleviated calcification of primary chondrocytes induced by FFSS

**Fig. 6:**
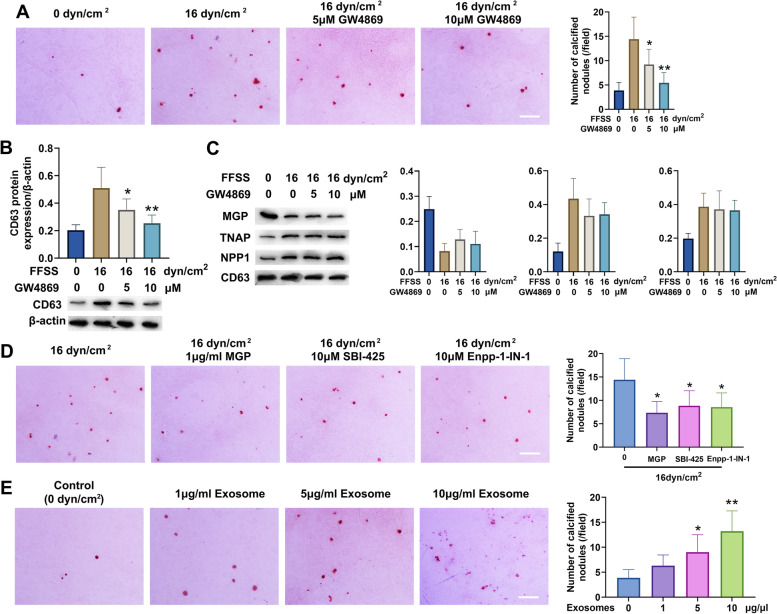
Inhibition of exosome formation alleviated calcification of condylar chondrocytes induced by FFSS. **A** Inhibition of exosome formation with GW4869 decreased the number of calcified nodules after FFSS stimulation in cultured condylar chondrocytes (*n* = 6). **B** GW4869 decreased exosome formation by condylar chondrocytes stimulated with FFSS (*n* = 6). **C** Inhibition of exosome formation did not affect the changes in protein levels in exosomes induced by FFSS (*n* = 6). Scale bar = 100 μm. All data are presented as means ± SD. Comparisons among groups were performed by one-way ANOVA, and it was followed by Tukey’s post hoc tests to evaluate the statistical significance in groups with the control group (0 dyn/cm^2^ group), respectively. *P<0.05, **P < 0.01, compared with the control group (0 dyn/cm^2^ group). **D** Supplementation with exogenous MGP or inhibition of TNAP and NPP1 effectively decreased the numbers of calcified nodules in condylar chondrocytes stimulated with FFSS (*n* = 6). Scale bar = 100 μm. Comparisons among groups were performed by one-way ANOVA, and it was followed by Tukey’s post hoc tests to evaluate the statistical significance in groups with the 16 dyn/cm^2^ group respectively. *P < 0.05, **P < 0.01, compared with the 16 dyn/cm^2^ group. **E** Exosomes from condylar chondrocytes stimulated with FFSS promoted the formation of calcified nodules in cultured condylar cartilage (*n* = 6). Scale bar = 100 μm. Comparisons among groups were performed by one-way ANOVA, and it was followed by Tukey’s post hoc tests to evaluate the statistical significance in groups with the control group (0 dyn/cm^2^ group), respectively. The data of compared groups were from the populations of Gaussian distribution and consistent with homogeneity of variance. *P < 0.05, **P < 0.01, compared with the control group (0 dyn/cm^2^ group)

Based on the above results, 16 dyn/cm^2^ FFSS was selected to stimulate primary condylar chondrocytes in subsequent studies. GW4869 of different concentrations was used to inhibit the formation of exosomes. Alizarin red staining showed that GW4869 significantly and dose-dependently reduced the number of calcified nodules that formed in primary condylar chondrocytes after FFSS stimulation (5 μM GW4869: *P *< 0.05, 10 μM GW4869: *P *< 0.01) (Fig. 6A). The protein level of CD63 was also significantly reduced by GW4869 (5 μM GW4869: *P *< 0.05, 10 μM GW4869: *P *< 0.01) (Fig. 6B). However, the protein levels of MGP, TNAP, and NPP1 in the secreted exosomes were not affected by the addition of GW4869 (Fig. 6C), suggesting that the inhibitory effect of GW4869 on calcification was achieved mainly via inhibition of exosome formation rather than via changes in the contents of exosomes.

To determine whether components in exosomes are involved in calcification, 16 dyn/cm^2^ FFSS was selected as the stimulation condition, and 1 μg/ml MGP, 10 μM SBI-425 (TNAP inhibitor), and 10 μM Ennp-1-IN-1 (NPP1 inhibitor) were administered to condylar chondrocytes respectively. Alizarin red staining showed that the numbers of calcified nodules in cultured TMJ chondrocytes were significantly reduced after the addition of MGP, SBI-425, and Ennp-1-IN-1 (1 μg/ml MGP: *P *< 0.05, 10 μm SBI-425: *P *< 0.05, 10 μm Ennp-1-IN-1: *P *< 0.01) (Fig. 6D), suggesting that corresponding effects on exosome protein components can also significantly inhibit the calcification of TMJ chondrocytes.

We also added exosomes, secreted by condylar chondrocytes under stimulation with 16 dyn/cm^2^ FFSS, to the medium of condylar chondrocytes cultured without FFSS stimulation at different concentrations. Alizarin red staining showed that the numbers of calcified nodules were significantly increased (5 μg/ml exosomes: *P *< 0.05, 10 μg/ml exosomes: *P *< 0.01) in a dose-dependent manner (Fig. 6E), suggesting that exosomes secreted by FFSS-stimulated condylar chondrocytes were able to promote calcification.

### Local injection of GW4869 inhibited calcification in the rat cartilage of TMJ OA

**Fig. 7:**
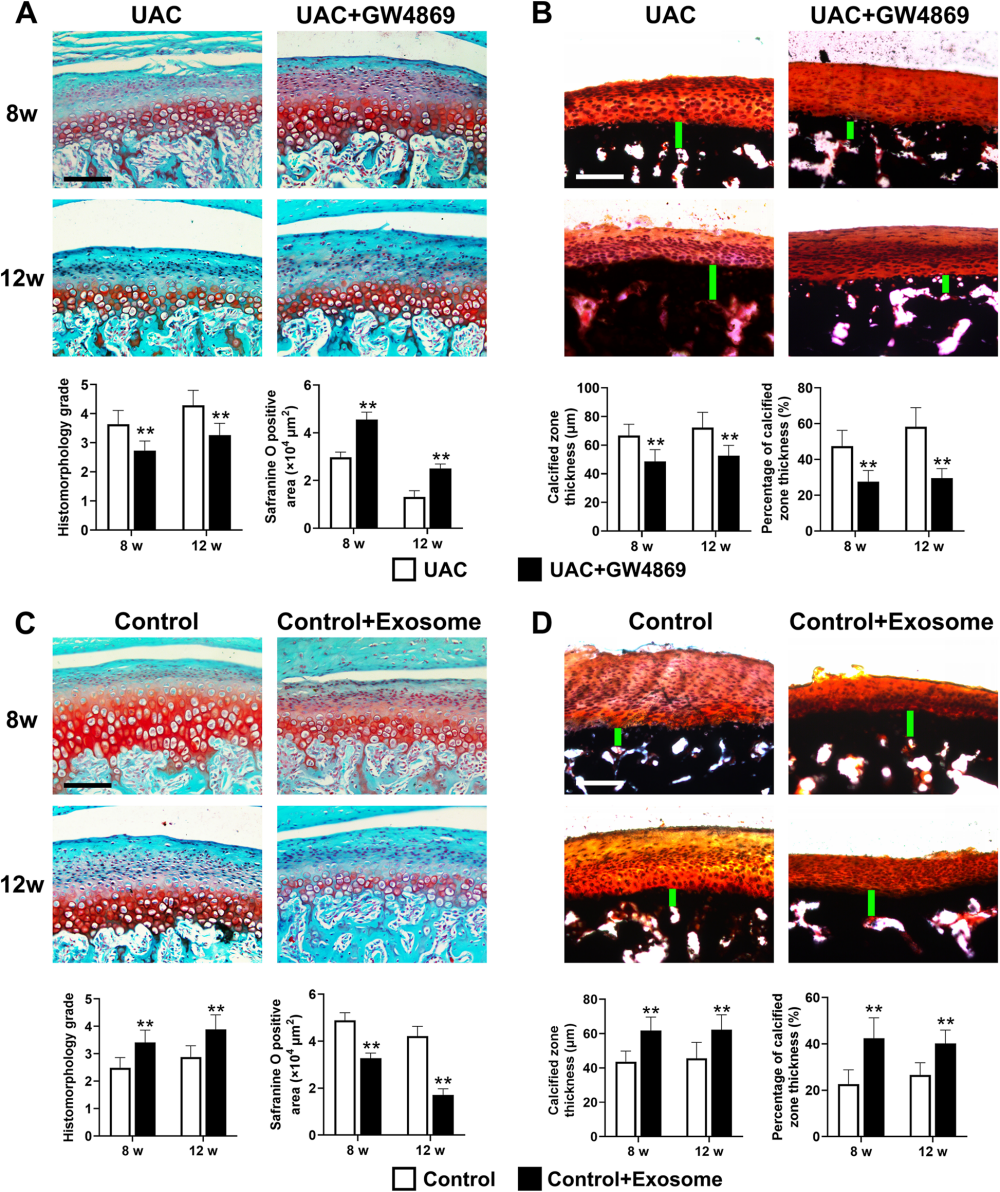
Effect of local injection of GW4869 and exosomes on cartilage degeneration and calcification of condylar cartilage in the TMJs of rats. **A** Cartilage degeneration of the TMJ was alleviated in UAC rats injected with GW4869 (*n* = 6). **B** Cartilage calcification of the TMJ was alleviated in UAC rats injected with GW4869 (*n* = 6). Scale bar = 100 μm. Green bar, calcified cartilage. UAC, unilateral anterior crossbite group; UAC + GW4869, unilateral anterior crossbite group with GW4869 injection; 8w, 8 weeks; 12w, 12 weeks. All data are presented as means ± SD. Comparisons between the UAC and UAC + GW4869 group of the same timepoint were performed by Student’s *t* test. **P < 0.01, compared with the age-matched UAC group. **C** Cartilage degeneration of the TMJ was induced in control rats with exosome injection (*n* = 6). **D** Cartilage calcification of the TMJ was induced in control rats with exosome injection (*n* = 6). Scale bar = 100 μm. Green bar, calcified cartilage. Control, control group; Control + Exosome, control group with exosome injection; 8w, 8 weeks; 12w, 12 weeks. All data are presented as means ± SD. The data of compared groups were from the populations of Gaussian distribution and consistent with homogeneity of variance. Comparisons between the control and control + exosome group of the same timepoint were performed by Student’s *t* test. **P < 0.01, compared with the age-matched control group

**Fig. 8:**
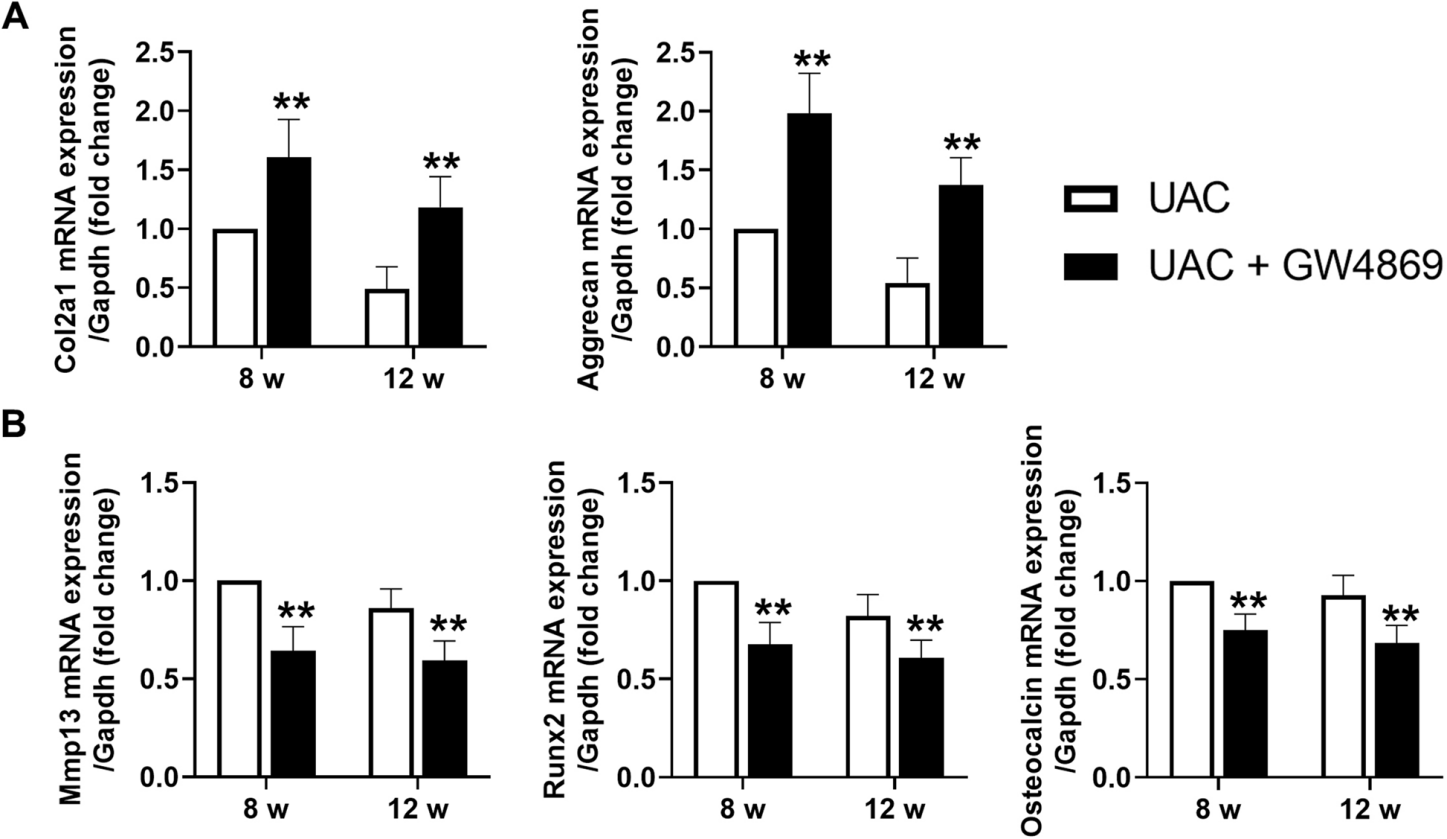
GW4869 injection reversed changed mRNA expression in condylar cartilage of rats with TMJ OA induced by UAC stimulation. **A** mRNA expression of Col2a1 and Aggrecan was significantly increased in the TMJs of UAC rats with GW4869 injection (*n* = 6). **B** mRNA expression of Mmp13, Runx2, and Osteocalcin was significantly decreased in the TMJs of UAC rats with GW4869 injection (*n* = 6). UAC, unilateral anterior crossbite group; UAC+GW4869, unilateral anterior crossbite group with GW4869 injection; 8w, 8 weeks; 12w, 12 weeks. All data are presented as means ± SD. The data of compared groups were from the populations of Gaussian distribution and consistent with homogeneity of variance. Comparisons between the UAC and UAC + GW4869 group of the same timepoint were performed by Student’s t-test. **P < 0.01, compared with the age-matched UAC group

To clarify the therapeutic effect of inhibition of exosome formation on cartilage calcification in TMJ OA, we locally injected exosomes into the TMJs of UAC rats. The safranine O-positive areas in condylar cartilage were significantly enlarged in GW4869-injected UAC rats compared with UAC-group rats (8 weeks: *P *< 0.01, 12 weeks: *P *< 0.01), indicating increased proteoglycan in the cartilage matrix. In addition, at 8 weeks and 12 weeks, the histomorphology grade of cartilage was significantly lower in the GW4869 injection groups (8 weeks: *P *< 0.01, 12 weeks: *P *< 0.01) (Fig. 7A). The thickness of the calcified layer in TMJ condylar cartilage (8 weeks: *P *< 0.01, 12 weeks: *P *< 0.01) and the proportion to the whole cartilage (8 weeks: *P *< 0.01, 12 weeks: *P *< 0.01) were significantly lower in the GW4869 injection groups than in the UAC groups (Fig. 7B). The mRNA expression of Col2a1, Aggrecan, Mmp13, Runx2, and Osteocalcin decreased all after GW4869 injection (Fig. 8).

**Fig. 9:**
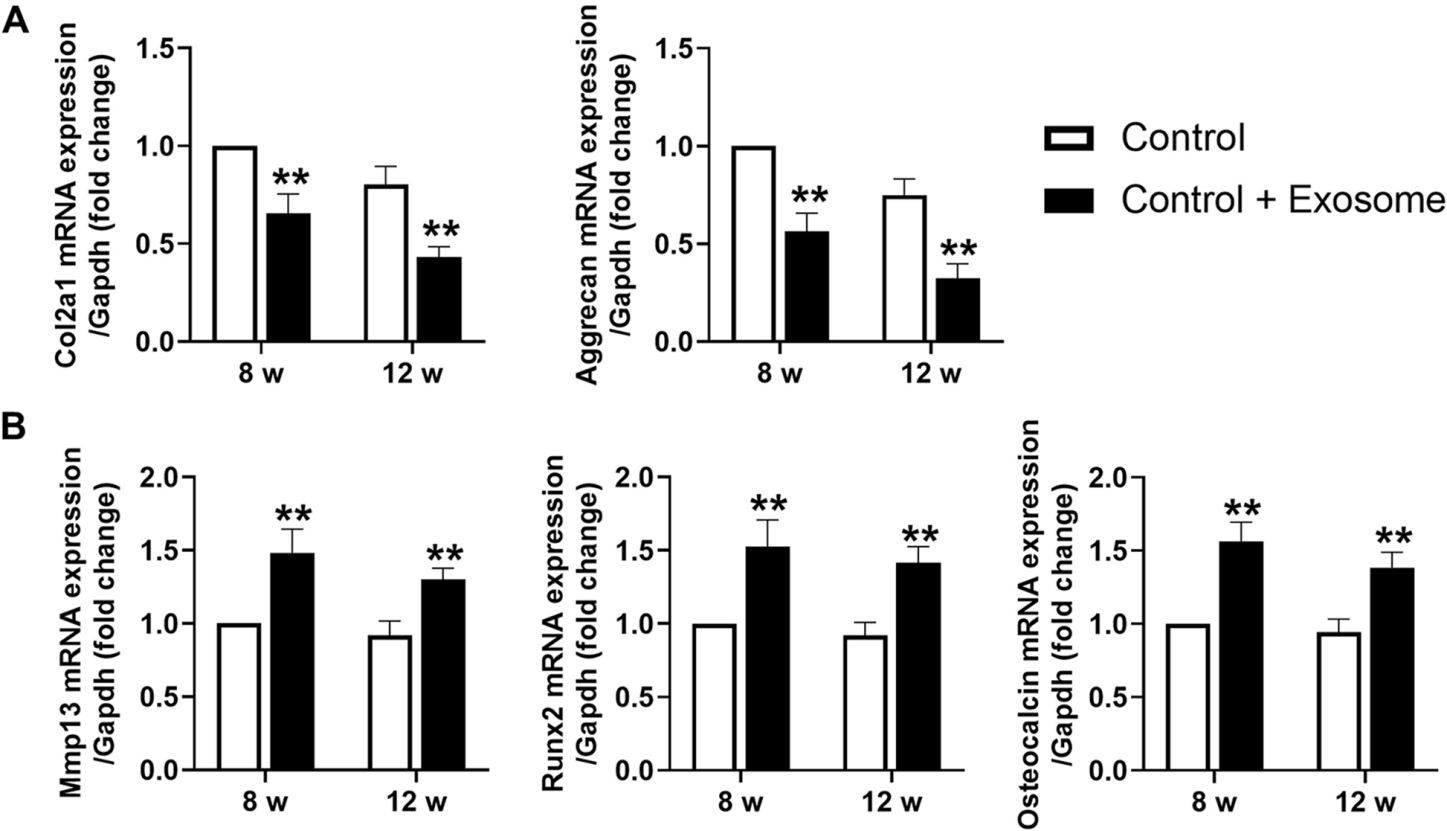
Exosome injection-induced degenerative lesion in condylar cartilage of control rats. **A** mRNA expression of Col2a1 and Aggrecan was significantly decreased in the TMJs of control rats with exosome injection (*n* = 6). **B** mRNA expression of Mmp13, Runx2, and Osteocalcin was significantly increased in the TMJs of control rats with exosome injection (*n* = 6). Control, control group; Control + Exosome, control group with exosome injection; 8w, 8 weeks; 12w, 12 weeks. All data are presented as means ± SD. The data of compared groups were from the populations of Gaussian distribution and consistent with homogeneity of variance. Comparisons between the control and control + exosome group of the same timepoint were performed by Student’s *t* test. **P < 0.01, compared with the age-matched control group

To verify the role of exosomes in promoting cartilage calcification of the TMJ in vivo, condylar chondrocytes were stimulated with 16 dyn/cm^2^ FFSS, and exosomes in the culture medium were collected and then locally injected into the TMJs of control rats. After injection of exosomes, the safranine O-positive area in condylar cartilage significantly decreased (8 weeks: *P *< 0.01, 12 weeks: *P *< 0.01), suggesting less proteoglycan in the cartilage matrix. In addition, at 8 weeks and 12 weeks, the histomorphology grade of cartilage in the exosome injection groups was also significantly increased (8 weeks: *P *< 0.01, 12 weeks: *P *< 0.01) (Fig. 7C). The thickness of the calcified layer in condylar cartilage (8 weeks: *P*<0.01, 12 weeks: *P*<0.01) and the proportion to the whole cartilage (8 weeks: *P *< 0.01, 12 weeks: *P *< 0.01) were higher in the exosome injection groups than that in the control groups (Fig. 7D). The mRNA expression of Col2a1 and Aggrecan was significantly decreased in the exosome injection groups, together with increased mRNA expression of Mmp13, Runx2, and Osteocalcin (Fig. 9).

We also tested the expression of pro-inflammatory cytokines, including TNF-α, IL-1β, IL-6, and IL-18, in the rat TMJ condylar cartilage in the groups of control, control with exosome injection, UAC, and UAC with exosome inhibitor injection at 12 weeks. The results showed that compared with the control group, the mRNA expression of pro-inflammatory cytokines in the control with exosome injection group and the UAC group was significantly increased (Tnf-a: *P *< 0.01, IL-1b: *P *< 0.01, IL-6: *P *< 0.001, and IL-18: *P *< 0.01). Meanwhile, the mRNA expression of pro-inflammatory cytokines in the UAC with exosome inhibitor group was significantly reduced compared with the UAC group (Tnf-a: *P *< 0.01, IL-1b: *P *< 0.05, IL-6: *P *< 0.001, and IL-18: *P *< 0.05) (Supplemental Fig. 2).

## Discussion

OA is a common degenerative disease of joints, and abnormal biomechanical force is one of the main causes of OA [27, 28]. We induced TMJ OA lesions by changing the occlusal relationship in rats to cause an abnormal biomechanical environment in the TMJs [5, 18]. Similar to knee osteoarthritic cartilage, the cartilage of TMJ OA exhibited not only degenerative changes, such as degraded matrix and reduced cartilage thickness, but also abnormal cartilage calcification, which manifested as increased thickness of calcified cartilage and an enlarged proportion of thickness of calcified cartilage to the full thickness of the cartilage [5, 19]. Our previous study confirmed that BCP crystals are the main calcium deposits in the abnormally calcified cartilage of TMJ OA and that BCP crystals can stimulate chondrocytes to produce tumor necrosis factor-α (TNF-α) interleukin-1β (IL-1β) and other proinflammatory factors, thus aggravating the inflammatory response of OA [5, 29]. In addition, BCP crystals can stimulate chondrocytes to produce matrix-degrading enzymes such as matrix metalloproteinase 3/9/13 and Adamts4/5, resulting in the destruction of the cartilage matrix [30, 31].

In the current study, TEM revealed that numerous exosomes were present around the chondrocytes in the abnormal calcified area of TMJ OA cartilage. In addition, the number of CD63-positive chondrocytes in this area was also increased significantly, suggesting that the increase in chondrocyte-derived exosome formation caused by abnormal biomechanical loading might induce abnormal calcification of cartilage. The in vitro experiments also showed that the formation of exosomes and the number of calcified nodules in cultured chondrocytes were significantly increased under FFSS stimulation. However, the numbers of calcified nodules decreased significantly after the use of GW4869 to inhibit exosome formation. Local injection of GW4869 into the joints of TMJ OA rats also reduced the abnormal calcification and degeneration of cartilage, whereas local injection of exosomes secreted by FFSS-stimulated chondrocytes into normal rats caused abnormal calcification and degeneration of TMJ cartilage. These results confirm that exosomes produced by chondrocytes stimulated with abnormal biomechanical forces are important regulators of abnormal calcification in cartilage. Thus, inhibition of exosome formation is expected to become a new target for OA treatment.

Exosomes contain large numbers of molecules [8]. At present, most studies on the functions of exosomes have focused mainly on the transmission of biological information, such as miRNA [32–34]. However, abnormal calcification in cartilage occurs mainly in the matrix, in which exosomes are secreted by chondrocytes, indicating that the secreted exosomes regulate calcification more directly through the proteins or enzymes they contain. Our results showed that FFSS stimulation not only increased the numbers of exosomes but also changed the levels of proteins in chondrocyte-derived exosomes; for example, it decreased the MGP content and increased the TNAP and NPP1 content.

MGP is found mainly in the extracellular matrix of cartilage, bone, and arterial vessel walls [35, 36]. MGP overexpression decreases mineralization in vitro, and deficient MGP expression impairs bone growth and induces ectopic mineralization in the arterial vessel walls of mice [37, 38]. Stimulation of human osteoarthritic chondrocytes with shear stress reduces MGP mRNA expression, providing a mechanism for enhanced mineralization due to mechanical loading [39]. Our study showed that the content of MGP was decreased significantly in exosomes from chondrocytes stimulated with FFSS. In addition, the numbers of MGP-positive chondrocytes and the mRNA expression of Mgp were significantly reduced in the rats in the UAC groups. These results suggest that stimulation with abnormal biomechanical force lessens the ability of chondrocytes to synthesize MGP, leading to decreased levels of MGP in exosomes secreted into the matrix. These exosomes are unable to function properly to inhibit calcification. NPP1, encoded by the Ennp1 gene, is a member of the nucleotide pyrophosphatase/phosphodiesterase family that catalyzes reactions to produce pyrophosphate (PPi) and AMP from extracellular nucleotide triphosphates [40]. TNAP hydrolyzes PPi to release phosphate (Pi), and Pi is needed for the formation of BCP crystals [41]. Therefore, NPP1 and TNAP act to control the presence of substrate during the mineralization stage [42, 43]. In the current study, the levels of NPP1 and TNAP were significantly increased in exosomes secreted by FFSS-stimulated chondrocytes; these exosomes could therefore produce large amounts of Pi in the matrix for the formation of BCP crystals and promote the generation of calcified nodules. However, the contents of NPP1- and TNAP-positive chondrocytes and the mRNA expression of these molecules in the TMJ cartilage of rats were significantly increased in the UAC groups. The highly expressed NPP1 and TNAP could be released into the matrix through exosomes to function.

Notably, application of the exosome formation inhibitor GW4869 to chondrocytes significantly reduced the occurrence of abnormal calcification induced by the FFSS-mediated increase in exosome formation, but the levels of MGP, TNAP, and NPP1 in exosomes were not significantly affected. This finding suggests that the reduction in abnormal cartilage calcification observed after local injection of GW4869 was due mainly to the decreases in the numbers of exosomes secreted by chondrocytes rather than to the changes in the levels of protein/enzyme components in the exosomes. Therefore, even if the composition of exosomes from chondrocytes stimulated by abnormal biomechanical force cannot be changed, inhibition of the formation of chondrocyte-derived exosomes can exert a positive effect by inhibiting the abnormal calcification of OA cartilage and thus alleviating OA lesions.

There is no doubt that pro-inflammatory changes also play a crucial role in the degeneration of OA cartilage [44, 45]. We also tested the expression of pro-inflammatory cytokines, including TNF-α, IL-1β, IL-6, and IL-18, in the rat TMJ condylar cartilage. The results indicated that the exosomes derived from abnormal biomechanics-induced chondrocytes could induce the inflammation in the cartilage, whereas the inhibition of exosome formation alleviated the inflammation in degenerative cartilage. Although these results cannot clearly clarify the interaction between exosomes formation/secretion and pro-inflammatory changes in osteoarthritic cartilage, it partially proves that chondrocyte-derived exosomes have influence on the expression of pro-inflammatory cytokines. The injection of exosomes produced by abnormal biomechanical force-stimulated chondrocytes to the TMJ of rats promoted the expression of inflammatory cytokines, while the injection exosome inhibitors to rats with cartilage degeneration in the UAC group reduced the expression of inflammatory cytokines. Moreover, the cause of these changes might be that injected exosomes or exosome inhibitors influenced abnormal calcification in cartilage, and in turn changed the biomechanical properties of cartilage, which further affected the expression of inflammatory cytokines in chondrocytes.

## Conclusion

Abnormal biomechanical loading decreases the calcification inhibitor (such as MGP) in TMJ condylar chondrocytes and increases the promotors (such as NPP1 and TNAP), thus changing the levels of related molecules in chondrocyte-derived exosomes. In addition, abnormal biomechanical loading promotes the formation and secretion of exosomes. Secreted exosomes promote calcification in the cartilage matrix. Inhibition of exosome formation can effectively alleviate abnormal calcification in degenerative cartilage in TMJ OA. The study provided the new insight in the influence of exosomes on osteoarthritic cartilage, revealed that exosomes not only induce inflammation changes in the process of OA, but also lead to abnormal calcification in cartilage, which bring about worse mechanical properties of cartilage and then intensified the destruction of cartilage in OA.

## Supplementary Information

**Additional file 1**: **Supplemental Table 1**. Gene primer sequences of rats used for qRT-PCR. **Supplemental Figure 1**. The representative central sagittal hematoxylin-eosin staining images of rat TMJ represents the outline of measurement for quantitative assessment of histological parameters. The image of condylar cartilage was divided into three sections with equal width (anterior, middle and posterior). The region of interest (ROI) at the center of each section was boxed (width: 200mm, height: cartilage thickness) and analyzed for the thickness of the cartilage or calcified cartilage. The values from three ROIs were averaged and reported for each sample. **Supplemental Figure 2**. Effect of local injection of GW4869 and exosomes on the inflammation in TMJ cartilage of rats. mRNA expression of Tnf-a, IL-1b, IL-6 and IL-18 in groups of control, control with exosome injection, UAC and UAC with exosome inhibitor injection at 12 w. Control, control group; Control + Exosome, control group with exosome injection; UAC, unilateral anterior crossbite group; UAC + GW4869, unilateral anterior crossbite group with GW4869 injection. All data are presented as means ± SD. The data of compared groups were from the populations of Gaussian distribution and consistent with homogeneity of variance. Comparisons between the control and control + exosome groups or UAC and UAC + GW4869 groups were performed by Student’s t-test. *P < 0.05, **P < 0.01 and ***P < 0.001.

## Data Availability

The datasets used and/or analyzed during the current study are available from the corresponding author on reasonable request.

## Abbreviations

TMJ: Temporomandibular joint
OA: Osteoarthritis
FFSS: Fluid flow shear stress
MGP: Matrix Gla protein
TNAP: Tissue-nonspecific alkaline phosphatase
NPP1: Nucleotide pyrophosphatase/phosphodiesterase-1
TMD: Temporomandibular disorder
BCP: Basic calcium phosphate
CPPD: Calcium pyrophosphate dihydrate
UAC: Unilateral anterior crossbite
TEM: Transmission electron microscopy
Col2a1: Type-II collagen
Mmp13: Matrix metallopeptidase 13
Runx2: Runt-related transcription factor 2
TNF-α: Tumor necrosis factor-α
IL-1β: Interleukin-1β
