# Supplementary material for: Chondrocyte-derived exosomes promote cartilage calcification in temporomandibular joint osteoarthritis
Source: Arthritis Res Ther. 2022 Feb 14;24:44. doi: 10.1186/s13075-022-02738-5 (PMC8842872; doi:10.1186/s13075-022-02738-5)
Supplement: Supplementary file 1 — Additional file 1: Supplemental Table 1. Gene primer sequences of rats used for qRT-PCR. Supplemental Figure 1. The representative central sagittal hematoxylin-eosin staining images of rat TMJ represents the outline of measurement for quantitative assessment of histological parameters. The image of condylar cartilage was divided into three sections with equal width (anterior, middle and posterior). The region of interest (ROI) at the center of each section was boxed (width: 200mm, height: cartilage thickness) and analyzed for the thickness of the cartilage or calcified cartilage. The values from three ROIs were averaged and reported for each sample. Supplemental Figure 2. Effect of local injection of GW4869 and exosomes on the inflammation in TMJ cartilage of rats. mRNA expression of Tnf-a, IL-1b, IL-6 and IL-18 in groups of control, control with exosome injection, UAC and UAC with exosome inhibitor injection at 12 w. Control, control group; Control + Exosome, control group with exosome injection; UAC, unilateral anterior crossbite group; UAC + GW4869, unilateral anterior crossbite group with GW4869 injection. All data are presented as means ± SD. The data of compared groups were from the populations of Gaussian distribution and consistent with homogeneity of variance. Comparisons between the control and control + exosome groups or UAC and UAC + GW4869 groups were performed by Student’s t-test. *P < 0.05, **P < 0.01 and ***P < 0.001. [file 13075_2022_2738_MOESM1_ESM.docx]

**Chondrocyte-derived exosomes promote cartilage calcification in temporomandibular joint osteoarthritis**

Qian Liu^1a^, Ruoxin Wang^2a^, Shujie Hou^3a^, Feng He^1^, Yuanjun Ma^1^, Tao Ye^1^, Shibin Yu^1^, Hongwei Chen^4^, Helin Wang^5*^, Mian Zhang^1*^

^1^State Key Laboratory of Military Stomatology & National Clinical Research Center for Oral Diseases & Shaanxi International Joint Research Center for Oral Diseases, Department of Oral Anatomy and Physiology and TMD, School of Stomatology, the Fourth Military Medical University, Xi’an, China

^2^Class 1, Grade 2018, School of Stomatology, Zhengzhou University, Zhengzhou, China

^3^School of Basic Medicine, the Fourth Military Medical University, Xi’an, China

^4^Health Center of 73630 Unit of the Chinese People's Liberation Army, Fuzhou, China

^5^State Key Laboratory of Military Stomatology & National Clinical Research Center for Oral Diseases，Department of Medical Rehabilitation, School of Stomatology, the Fourth Military Medical University, Xi’an, China

^a^Authors contributing equally to this work.

***Corresponding Authors:**

Mian Zhang, State Key Laboratory of Military Stomatology & National Clinical Research Center for Oral Diseases & Shaanxi International Joint Research Center for Oral Diseases, Department of Oral Anatomy and Physiology and TMD, School of Stomatology, Fourth Military Medical University, Changle West Road, Xi’an, 710032, China. Email: zhangmian1986@aliyun.com

Helin Wang, State Key Laboratory of Military Stomatology & National Clinical Research Center for Oral Diseases，Department of Medical Rehabilitation, School of Stomatology, the Fourth Military Medical University, Changle West Road, Xi’an, 710032, China. Email: 921441502@qq.com

**Supplemental Table 1. Gene primer sequences of rats used for qRT-PCR**

| Genes | Forward primer | Reverse primer |
| --- | --- | --- |
| Tnf-a | ATGGGCTCCCTCTCATCAGTTCC | CCTCCGCTTGGTGGTTTGCTAC |
| IL-1b | AATCTCACAGCAGCATCTCGACAAG | TCCACGGGCAAGACATAGGTAGC |
| IL-6 | ACTTCCAGCCAGTTGCCTTCTTG | TGGTCTGTTGTGGGTGGTATCCTC |
| IL-18 | CGACCGAACAGCCAACGAATCC | GTCACAGCCAGTCCTCTTACTTCAC |


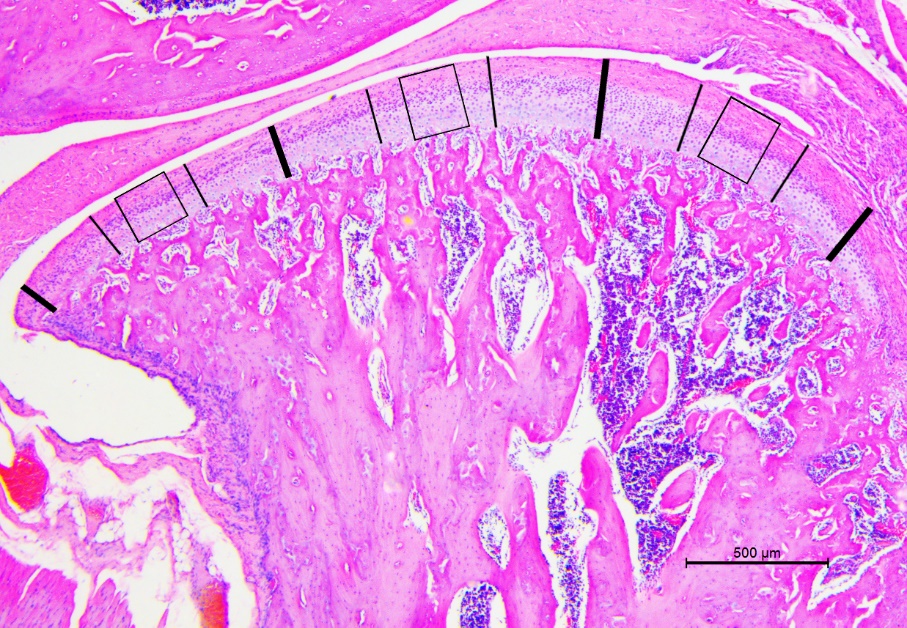


Supplemental Fig1. The representative central sagittal hematoxylin-eosin staining images of rat TMJ represents the outline of measurement for quantitative assessment of histological parameters. The image of condylar cartilage was divided into three sections with equal width (anterior, middle and posterior). The region of interest (ROI) at the center of each section was boxed (width: 200mm, height: cartilage thickness) and analyzed for the thickness of the cartilage or calcified cartilage. The values from three ROIs were averaged and reported for each sample.


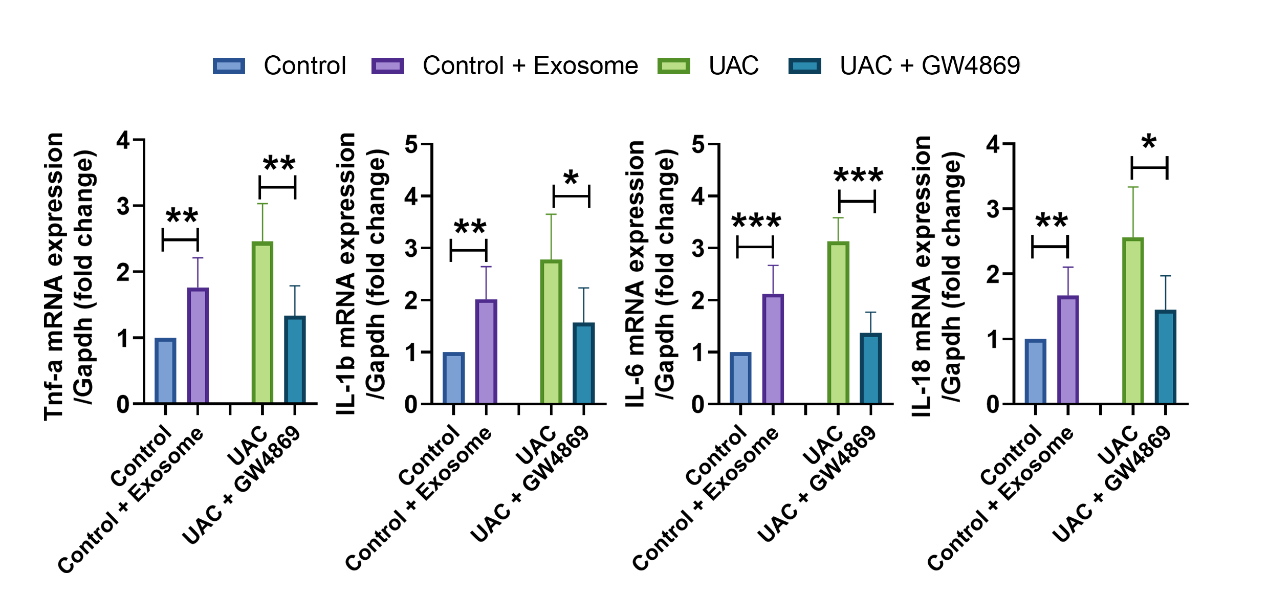


Supplemental Fig2. Effect of local injection of GW4869 and exosomes on the inflammation in TMJ cartilage of rats. mRNA expression of Tnf-a, IL-1b, IL-6 and IL-18 in groups of control, control with exosome injection, UAC and UAC with exosome inhibitor injection at 12 w. Control, control group; Control + Exosome, control group with exosome injection; UAC, unilateral anterior crossbite group; UAC + GW4869, unilateral anterior crossbite group with GW4869 injection. All data are presented as means ± SD. The data of compared groups were from the populations of Gaussian distribution and consistent with homogeneity of variance. Comparisons between the control and control + exosome groups or UAC and UAC + GW4869 groups were performed by Student’s t-test. *P<0.05, **P<0.01 and ***P<0.001.
